# The Professional Spirit

**Published:** 1903-04-15

**Authors:** C. N. Johnson


### THE PROFESSIONAL SPIRIT.

BY C. N. JOHNSON, L.D.S., D.D.S.

It would sometimes seem, in the intense activity of thought and effort along material lines mainfest in our ranks to-day, that the spirit of true professionalism was in danger of being lost to view. Men are apt to forget the higher essence of their calling when their energies are directed and their attention absorbed in the one aim of materialistic advancement, and there comes a time when it appears necessary to call a halt and take serious stock of our real position and see whether or not we are living up to the best traditions of our craft.

It seems difficult to instill into the minds of many of those practicing dentistry the true significance of what professionalism really is. To be blunt about it, the fact is, a very appreciable number of men in the profession to-day were not born right to begin with. That is, they were not born right for professional men. They might have made good mechanics, or good tailors, or good barbers, or good stonemasons, or good fellows—but not good professional men. It may be added in parenthesis, and without the slightest breach of confidence, that some of them might have made good politicians. But to be a professional man in the truest sense is something apart from all this. The essence of real professionalism must either be bred in the bone, or at least nurtured assiduously in the development of the individual. Say what we may in our dubious moments, there is a vast distinction between the practice of a profession and the following of a trade or commercial pursuit, though it is true that many in our ranks apparently fail to realize this distinction.

### PROFESSIONALISM IN THE COLLEGE COURSE.

The reason that an offensive commercialism is too often found associated with dental practice is because many dentists lack the true professional spirit. They were neither born with the professional instinct nor has their education imparted to them the professional idea. And just here I must pause to consider what I believe to be one of the most important factors connected with the development of professionalism in dentistry. To-day it is safe to say that ninety percent of those entering the profession receive their early impressions of professional matters through the medium of dental colleges. The number of those seeking admission through other sources than a college course is almost infinitesimal, and so we must look to the college to impart the professional idea of the coming dentist. I am not one of those who flippantly lay all the sins of professional commission or omission at the doors of the colleges, because my association with college men has led me to believe that they are an earnest, conscientious, hard-working body of men. There are black sheep in every fold, but that the large majority of those engaged in teaching in our dental colleges to-day are as lacking in principle as has sometimes been charged I am not quite prepared to accept. And yet it has occasionally occurred to me that it was possible for college men to accomplish more than they are now doing in the way of building up a professional spirit among their graduates. The intense activity and the rapid evolution of methods manifest in teaching students the fundamental and practical branches of dentistry in the last few years have occupied the best energies of the best men to keep pace with the advancement, and if the less tangible though higher phases of mental and moral development have not always received their due consideration, it is not altogether to be wondered at. It is true that every well-appointed college has a series of lectures on ethics, and also high moral principles are sought to be inculcated into the student body at all times, but this is usually only in a general way and not with the specific aim of developing that finer sense of true professionalism which should enter the very life, and color the conduct of every practitioner of dentistry.

### DUTY OF THE TEACHER.

College teachers should constantly study their students with the one aim of making professional gentlemen of them, and, with this end in view, they should never lose an opportunity to emphasize their professional obligations and to point out the true significance of what professionalism implies. It goes without saying that in order to accomplish anything along this line with a class of students the teacher himself must have a just appreciation of the dignity of his calling, and be a man of the highest moral and mental tone. He must at all times so tinge his remarks that the students are early impressed with the fact that in the study of dentistry they are assuming a different species of obligation from the man learning a trade or following a commercial pursuit. If the teachers in all of our colleges will approach their work with the proper professional spirit themselves, it will in the end do much toward developing such a spirit in the rank and file of the profession.

But it may be well to consider for a moment what is involved in the obligations of a professional man aside from those of a tradesman. The one great distinction, as I view it, relates to the question of free service to humanity. It is seldom or never binding on a man engaged in a trade to give his services free of charge, while with the professional man—particularly those associated with the healing art—it is frequently necessary to exert his very best efforts with absolutely no remuneration. A dentist or a physician is obliged at all times to relieve suffering wherever or whenever found entirely regardless of any possible financial return. If a poor mortal is racked with pain, whether he be prince or pauper, the true professional man must use his best skill to relieve the pain without a thought as to any possible reward. This is one of the unwritten obligations with which he binds himself when he elects to follow a profession. The moment he assumes professional status he places himself in such a relationship to humanity at large that the poorest creature in existence has that claim upon him. It was formerly the rule that the professional man performed his work without even estimating financially the value of his services. He simply relieved those in need of his aid as a matter of course, and in the event of receiving remuneration it came to him voluntarily from the one relieved as an honorarium. This was particularly true in the old days of serfdom, when the slaves of an estate received the same professional care as the master, and at stated times—usually at the end of the year—the master sent his professional man his regular stipend, not as a transaction of barter, but as a voluntary honorary offering. The abolishment of serfdom and the general leveling up of the different classes of society changed the conditions so that a different system became necessary. Under the old system with the new conditions the livelihood of the professional man became too precarious, and so in his own self-defence he was obliged to make a definite charge for his services. This was only proper and just, and yet in this introduction of the commercial element into the profession there was something of the old spirit of true professionalism lost, 'till to-day the trend in the professions seems dangerously near drifting solely in the direction of commercialism to the utter exclusion of the professional.

### BUSINESS AND PROFESSIONALISM.

It is, of course, in strict accordance with equity and justice that a man, professional or otherwise, should gather together sufficient of the world's material goods to insure himself and those dependent upon him from want in old age. In fact, this is something more than his right; it is a solemn and moral duty. But in assuming this common and manifest obligation of life he need not, as many seem to do, lose sight of the higher aspirations which should dominate every man's motives when he assumes to take rank in the world as a member of one of the learned professions. The very learning which he is supposed to possess places him in a relationship to humanity which distinguishes him from other men. The knowledge of the tradesman is more in the nature of common or ordinary knowledge, is not necessarily intricate, mysterious, or unfathomable by the average individual, while that possessed by the professional man is more specialized, profound, and what may be termed extraordinary. It is, essentially, knowledge which is not available to the rank and file.

Then, again, another distinction between the tradesman and the professional man is that the former deals with things while the latter deals with persons. If a stonemason lays a brick out of line or a carpenter swings a door the wrong way the mistake is readily recognized by any one and the error is not fundamentally disastrous to the welfare of mankind; but let a physician prescribe the wrong drug or a dentist maltreat an alveolar abscess, and no ordinary individual can detect the mistake, while the effect may easily be the jeopardy of human life. This does not imply that the tradesman is not a necessary and useful member of society and entitled to every respect for his skill and utility in performing the various offices assigned to him in the great machinery of the world's work, but merely that the nature of his obligations to humanity is not of the same specialized and intimate character as that of the professional man, nor are his responsibilities so great.

Looking at it from this point of view, it will be seen that the professional man is in need of a different sentiment and that he should be dominated by different impulses from men in other walks of life, and to establish this idea it is necessary to develop in the professional, as the bone and sinew of our professional faith, a spirit of broad humanitarianism which looks only to the greatest good for the greatest number, and which rigidly excludes selfishness of motive or narrowness of purpose.

### IMPORTANCE OF ASSOCIATIONS.

To be more specific as to what the true professional spirit may accomplish for us and what its absence may entail upon us, it is only necessary to consider briefly some of the signs of the times in the profession to-day, and study the tendency in the professional lives of some of our members. It must be acknowledged that one of the most potent factors in professional progress is the establishment and maintenance of professional associations. Dental societies have done more for dentistry than has any other one element in its advancement, and they have unique possibilities for the development and fostering of the professional spirit. But do dental societies always fulfill their highest function as instruments of good in this respect? Some societies undoubtedly do, but many others do not. And to what must we attribute the fact that society work is not always productive of the greatest possible benefit? Mainly to the one factor of personal ambition on the part of the members.

### POLITICAL AMBITION.

Ambition is a most excellent thing in a man when it is directed solely to the accomplishment of worthy measures, but when it takes the form of personal aggrandizement, and an itching for office, it is productive only of evil. To be plain about the matter, the influence of politics has done more harm to dentistry than can well be imagined. It does not seem to be enough on the part of some men engaged in society work to take an active and altogether laudable interest in the election to office of men who are capable of conducting the affairs of the organization in a creditable manner, but they must needs stoop to the methods of ward heelers and disreputable political tricksters, to the end they often convert what is supposed to be a dignified professional meeting into the turmoil of a political fracas. And what is it all for? I confess to as full an appreciation as any of the honor and distinction which comes to a man as the result of high office conferred upon him by his associates —provided it comes in the right way. If a man is made president of his society by virtue of a spontaneous recognition of his worth and fitness for the place, and because he is entitled to it from long service, he may well take a pardonable pride in its acceptance; but if the office carries with it the reeking odor of base political intrigue and dishonest methods, he would better far have a mill-stone hanged about his neck than that he should accept such questionable distinction.

And, after all, what is there in the fleeting honor of office to make men stoop so low to obtain it? I often marvel at the egregious folly of men in the profession who work harder at politics than they do to further the scientific interests of their calling. The only lasting reputation a man can make in the profession is to do something to lighten up the dark places in its scientific knowledge, and if all of the energy heretofore worse than wasted on politics had been expended in this direction the profession would have been vastly the better for it. Politics and professionalism are as far apart as the poles, and no one thing has done more to jeopardize the good of societies than has the too frequent introduction of politics into the meetings. Not only this, but it has so tinged the thought of the membership that the spirit of professionalism, instead of growing as it should in this most fertile field, has been choked out and well-nigh smothered by the noxious weed.

The possibilities of the professional spirit in the upbuilding of dentistry cannot be well computed. It is this which imparts to men the impulse to work unselfishly for the common good, to dig out the hidden mysteries of the forces of nature and apply them to the betterment of the profession. It makes a man study for hours in the quiet of his laboratory, late into the night and early in the morning, that he may learn something which shall be of benefit to his fellow-man. And if he is a true professional man he does it not for the glory it brings, not for the plaudits of the the passing crowd, not that his name may be heralded from one end of the land to the other, but simply that the knowledge he acquires may make the way clearer to his professional associates, and through them may lead to the amelioration of human suffering. The man who works solely for the purpose of making a reputation for himself, no matter how faithfully, or earnestly, or consistently—no matter what the sum total of his achievement may be—has not within him the true professional spirit. Reputation may come—it often does to the man who toils unselfishly for the public good—but the mainspring of his effort must not be impelled by personal aggrandizement, else the purest essence of his work is to that degree tarnished.

### INFLUENCE OF PROFESSIONAL SPIRIT ON CHARACTER.

The professional spirit is broadening in its tendency and takes a man out of the narrow rut of prejudice and self-conceit. It bids him acknowledge merit wherever he finds it and to give credit wherever credit is due. It makes him welcome the light, no matter by whom it is shed, and to accept a fact even if presented by his worst enemy. The greatest courage I can conceive of in any man is to frankly acknowledge his error when that error is pointed out by some one in opposition to him, and this rare quality in human nature is unerringly imparted to a man by the principle of professional spirit.

Years ago in ancient Galilee we had presented to us the example of a man who gave to this era its present name. Laying aside for the moment the religious sentiment with which his career is usually associated, I am impelled to point him out as the man of all others in the history of the world who typifies what I should like to conceive as the real professional spirit. His example in the attitude he assumed toward his fellow-man is something for us to study and to emulate. Not that you or I can ever hope to tune our hearts to the sublime sentiment which filled his soul, not that we can rise entirely superior to the small things of life and bear with such infinite patience as he the buffeting of the motley mob, not that you or I may assume to be in any degree molded in his master mind, but simply that we may with the example of his patience, his humility, his forbearance, and his loving kindness be pointed to the main essentials of that which I have feebly endeavored to portray as the true professional spirit.—*Cosmos.*

